# Phar-LSTM: a pharmacological representation-based LSTM network for drug–drug interaction extraction

**DOI:** 10.7717/peerj.16606

**Published:** 2023-12-14

**Authors:** Mingqing Huang, Zhenchao Jiang, Shun Guo

**Affiliations:** 1 School of Software Engineering, Shenzhen Institute of Information Technology, Shenzhen, Guangdong, China; 2 Shenzhen Institute of Advanced Technology, Chinese Academy of Sciences, Shenzhen, Guangdong, China

**Keywords:** Pharmacological representation, Long short-term memory, Multi-task learning, Drug–drug interaction extraction

## Abstract

Pharmacological drug interactions are among the most common causes of medication errors. Many different methods have been proposed to extract drug–drug interactions from the literature to reduce medication errors over the last few years. However, the performance of these methods can be further improved. In this paper, we present a Pharmacological representation-based Long Short-Term Memory (LSTM) network named Phar-LSTM. In this method, a novel embedding strategy is proposed to extract pharmacological representations from the biomedical literature, and the information related to the target drug is considered. Then, an LSTM-based multi-task learning scheme is introduced to extract features from the different but related tasks according to their corresponding pharmacological representations. Finally, the extracted features are fed to the SoftMax classifier of the corresponding task. Experimental results on the DDIExtraction 2011 and DDIExtraction 2013 corpuses show that the performance of Phar-LSTM is competitive compared with other state-of-the-art methods. Our Python implementation and the corresponding data of Phar-LSTM are available by using the DOI 10.5281/zenodo.8249384.

## Introduction

Identifying unknown drug interactions is of great benefit for the early detection of adverse drug reactions. In Europe and the USA, adverse drug reactions cause about 300,000 deaths annually (Zhang, Leng & Liu, 2020). A Drug–Drug Interaction (DDI) is a situation in which the effects of one drug are changed by the presence of another drug, and it is an important subset of adverse drug reactions (Brown & Winterstein, 2019; Lin et al., 2022; Cao et al., 2021; Karbownik et al., 2020). Therefore, detecting DDIs from the biomedical literature can be of great benefit for public health safety.

DDI extraction tasks can be typically divided into coarse-grained tasks and fine-grained tasks. A coarse-grained task aims to predict whether a pair of target drugs has a DDI, whereas a fine-grained task further distinguishes the specific type of the DDI. To address the DDI extraction problem, several platforms, such as the DDIExtraction 2011 (coarse-grained task) (Segura-Bedmar, Martínez Fernández & Sánchez Cisneros, 2011) and DDIExtraction 2013 (fine-grained task) (Segura-Bedmar, Martínez Fernández & Herrero Zazo, 2013) challenges have been proposed for evaluating the DDI extraction performance of different methods.

In recent years, various methods (Asada, Miwa & Sasaki, 2017; Björne, Kaewphan & Salakoski, 2013; Bobić, Fluck & Hofmann, 2013; Bokharaeian & Díaz, 2013; Chowdhury & Lavelli, 2013; Sánchez Cisneros, 2013; Hailu, Hunter & Cohen, 2013; Huang et al., 2017; Jiang, Gu & Jiang, 2017; Qian et al., 2022; Liu et al., 2016; Rastegar-Mojarad, Boyce & Prasad, 2013; Sahu & Anand, 2018; Kim et al., 2015; Thomas et al., 2013; Zhao et al., 2016; Chen et al., 2016b; Chen et al., 2016a; Chen et al., 2020; Peng et al., 2022) have been developed for DDI extraction. These studies can be roughly divided into two periods: The support vector machine (SVM) period and the deep learning period.

Before 2016, most methods were based on SVMs and focused on feature engineering and kernel crafting (Björne, Kaewphan & Salakoski, 2013; Bobić, Fluck & Hofmann, 2013; Bokharaeian & Díaz, 2013; Chowdhury & Lavelli, 2013; Sánchez Cisneros, 2013; Hailu, Hunter & Cohen, 2013; Rastegar-Mojarad, Boyce & Prasad, 2013; Thomas et al., 2013). For example, FBK-irst (Chowdhury & Lavelli, 2013) is a two-stage method that employs a hybrid kernel to detect DDIs and then assign each of the DDIs to one of the four types, wherein the hybrid kernel makes use of shallow linguistic information, a syntactic tree, and manually defined features. Kim et al. (2015) proposed a two-stage method based on a linear SVM that used rich features, such as a word feature, word-pair feature, parse-tree feature, and noun phrase constrained on coordination feature. NLLSS (Chen et al., 2016b) predicts potential synergistic drug combinations by integrating various types of information, including known synergistic drug combinations, drug-target interactions, and drug chemical structures, thereby enhancing treatment efficacy and reducing the need for high drug dosages to mitigate toxicity. Chen et al. (2016a) explored the future directions of network-based drug discovery and the network approach for personalized drug discovery by summarizing databases and web servers involved in drug-target identification and drug discovery processes. One main limitation of these methods is that their performance is largely dependent on the choice of the features.

After 2016, many deep learning-based methods were proposed to automatically extract the feature representations instead of manual feature engineering. Convolutional neural networks (CNNs) and Long Short-Term Memory networks (LSTMs) have been extensively applied by researchers. Representative CNN-based methods include naïve CNN (Liu et al., 2016), two-stage syntactic CNN (Zhao et al., 2016), and Attention CNN (Asada, Miwa & Sasaki, 2017). With respect to LSTM-based methods, many different models have been proposed, such as joint AB-LSTM (Sahu & Anand, 2018), two-stage LSTM (Huang et al., 2017), Skeleton-LSTM (Jiang, Gu & Jiang, 2017), and Attentive LSTM (Qian et al., 2022). By reviewing four experimental techniques utilized in recent years to search for small-molecule inhibitors of miRNAs, as well as three distinct models for predicting small molecule-miRNA associations from various perspectives, Chen et al. (2020) explored significant publicly accessible databases and web servers containing experimentally validated or potential associations. DAESTB (Peng et al., 2022) introduces a cutting-edge computational method for predicting associations between small molecules and miRNAs. This innovative approach integrates small molecule–small molecule similarity, miRNA–miRNA similarity, and known small molecule–miRNA associations into a high-dimensional feature matrix, leveraging a deep autoencoder and a scalable tree boosting model. Generally, these deep learning-based methods achieve higher performance than traditional SVM-based methods while requiring fewer handcrafted features. Many of these methods adopt the embedding strategy (*i.e.,* map the text information to high-dimensional vectors) to obtain the latent features from the biomedical literature, and this has been proved to be helpful in improving the DDI extraction performance. For instance, AB-LSTM (Sahu & Anand, 2018) uses word and position embedding, and two-stage LSTM (Huang et al., 2017) combines word embedding with part of speech tag embedding in the model. SCNN (Zhao et al., 2016) proposed a syntax word embedding strategy, in which information about the position and part of speech features was taken into account. However, these embedding strategies typically ignore the information associated with the target drug, which would be conductive for more accurate extraction of DDIs.

In this article, we present a novel pharmacological representation-based long short-term memory network, named Phar-LSTM, for DDI extraction. The main contributions of this article are summarized as follows:

(1) A newly defined embedding strategy is proposed to extract pharmacological representations from the biomedical literature by combining word embedding with target drug related information embedding (*e.g.*, embedding the degree of the correlation between the word and the target drug, the relative position information of the target drug for each word) in our model.
(2) An LSTM-based multi-task learning scheme is introduced to jointly tackle the related tasks of DDI extraction (*i.e.,* determine whether the given document contains a DDI and identify the specific DDI types) and capture the common features that would benefit both tasks.
(3) We explore the DDI extraction performance of the models with 10 different LSTM variants.
(4) Experiments on the DDIExtraction 2011 and DDIExtraction 2013 corpuses were conducted to evaluate the performance of the proposed method, and the results show that our method outperforms other state-of-the-art methods on both datasets.

## Materials and Methods

**Figure 1:**
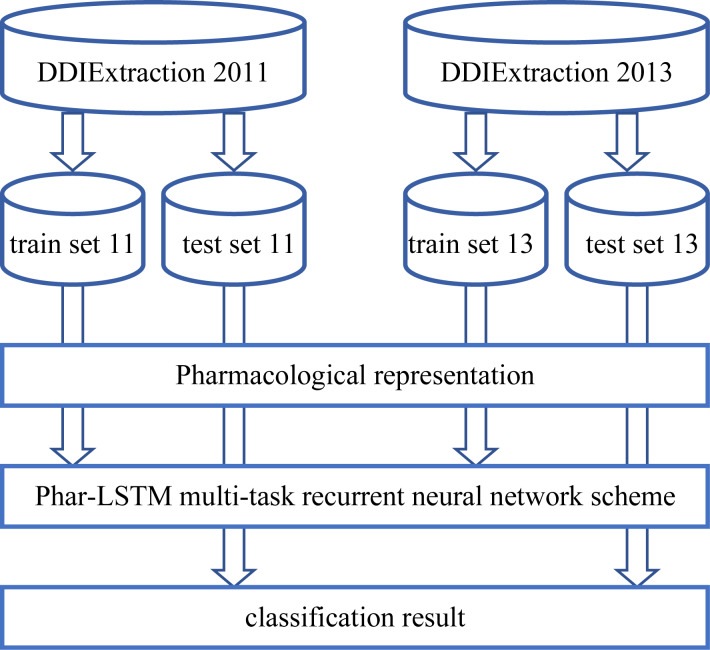
Overall processing flow of our Phar-LSTM scheme.

The overall process of our Phar-LSTM method is composed of three parts (illustrated in Fig. 1): (1) Extracting the pharmacological representations from the datasets, which consists of different but related tasks according to the newly defined embedding strategy; (2) taking the pharmacological representations as the input, and extracting the common features of the related tasks through the LSTM-based multi-task learning scheme; (3) the shared features are fed to the corresponding classifier for each task, and the classification results are regarded as the final output.

### Datasets

Text mining and natural language processing have recently benefitted the pharmacological industry. The DDIExtraction 2011 (Segura-Bedmar, Martínez Fernández & Sánchez Cisneros, 2011) and DDIExtraction 2013 (Segura-Bedmar, Martínez Fernández & Herrero Zazo, 2013) challenge tasks are held to promote the research of DDI extraction by providing benchmark datasets and enabling researchers to compare their methods fairly. The DDIExtraction 2011 challenge focuses on the binary classification of DDIs, that is, deciding whether the given document contains DDIs. For the DDIExtraction 2013 challenge, researchers must identify five DDI types: ADVICE, EFFECT, MECHANISM, INT, and NEGATIVE, which can be considered to be a multi-class classification problem.

The DDIExtraction 2011 dataset includes 579 documents about 14,949 drugs from DrugBank. These DrugBank documents contain rich chemical and pharmaceutical information. There are 5,806 sentences containing 3,160 DDIs (binary) in the DDIExtraction 2011 dataset. The DDIExtraction 2013 dataset has 784 documents from DrugBank and 233 abstract documents from MedLine, with a total of 5,021 DDIs (five specific DDI types). We selected task 9.2 as the testing dataset. More details of the datasets, including the training and testing information, can be found in Segura-Bedmar, Martínez Fernández & Sánchez Cisneros (2011) and Segura-Bedmar, Martínez Fernández & Herrero Zazo (2013).

### Embedding based pharmacological representation

To obtain useful pharmacological information from the biomedical literature, we present a newly defined embedding strategy to convert the raw input (biomedical documents) into high-dimensional vectors, that is, the pharmacological representations. It should be noted that a major difference between DDI extraction and other natural language processing tasks is that the two target drug entities in the DDI instance should be fully considered since the target drug pair contains important pharmacological information. For a document containing *n* drugs, there are ${C}_{n}^{2}$ DDI candidates. A document may contain more than one DDI instance, and all DDI candidates in the same document are expected to differ from each other. A common way to represent a DDI is “drug blinding”, that is, replacing the two target drugs with “drug1” and “drug2”, and the other drugs in the document are represented as “durg0” (Liu et al., 2016). However, this drug blinding strategy may discard some valuable pharmacological information contained in the target drugs (*e.g.*, the distinguishing information between different target drugs).

**Figure 2:**
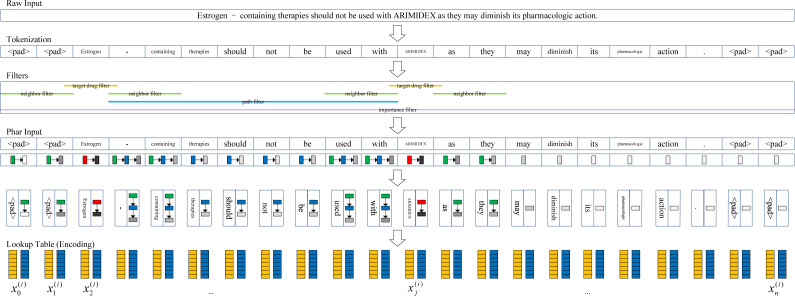
Pharmacological representation framework.

The pharmacological representation framework is shown in Fig. 2. First, we tokenized the documents into token sequences. It should be noted that different from the drug blinding strategy, the target drugs are not replaced with the words “drug1” and “drug2”. Therefore, more pharmacological information is extracted. The *t* th token unit was mapped to the ${x}_{t}^{(\mathrm{token})}$ using the word embedding strategy. We transformed each token into a *d* dimensional vector through random encoding as inspired by Wieting & Kiela (2019), wherein the values for each dimension are in $ \left[ -1/\sqrt{d},1/\sqrt{d} \right] $. In practice, we set *d* = 400 in our experiments.

Second, to extract the target drug related information, a set of filters are introduced to obtain the corresponding information in four aspects from the token sentence. The target drug filter determines whether a token is a target drug or not (1: True; 0: False), and the neighbor filter determines whether a token is a neighbor of the target drug (1: True; 0: False). Whether a token exists between a pair of target drugs (1: True; 0: False) is determined by the path filter. The importance filter measures the degree of the token associated with the nearest target drug. The closer the distance, the higher the degree (*i.e.,* more importance is attached).

We define the metric *I* = 1/(*r* + 1)^2^, where *r* is the distance from the token to the nearest target drug (*e.g.*, if the token is the target drug, *r* = 0; if the token is located near to the target drug, *r* = 1). Similar to the vector space model (Salton, Wong & Yang, 1975), we characterize the target drug related information of the *t* th token as a four-dimensional vector ${x}_{t}^{(\mathrm{Phar})}$.

Finally, a given document $D= \left( {w}_{0},{w}_{1},\ldots ,{w}_{m} \right) $ with *m* words is represented by ${D}^{(\mathrm{input})}=(({x}_{0}^{(\mathrm{token})},{x}_{0}^{(\mathrm{Phar})}),({x}_{1}^{(\mathrm{token})},{x}_{1}^{(\mathrm{Phar})}),\ldots ,({x}_{n}^{(\mathrm{token})},{x}_{n}^{(\mathrm{Phar})}))$, which we call pharmacological representation. Usually, *n* is larger than *m* because a document may be tokenized by splitting using punctuation, such as “-” and “.”.

### LSTM-based multi-task learning

**Figure 3:**
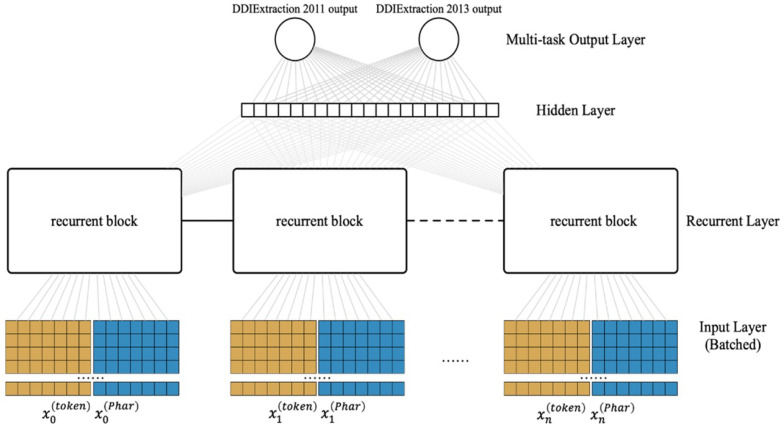
Architecture of our LSTM-based multi-task learning.

**Figure 4:**
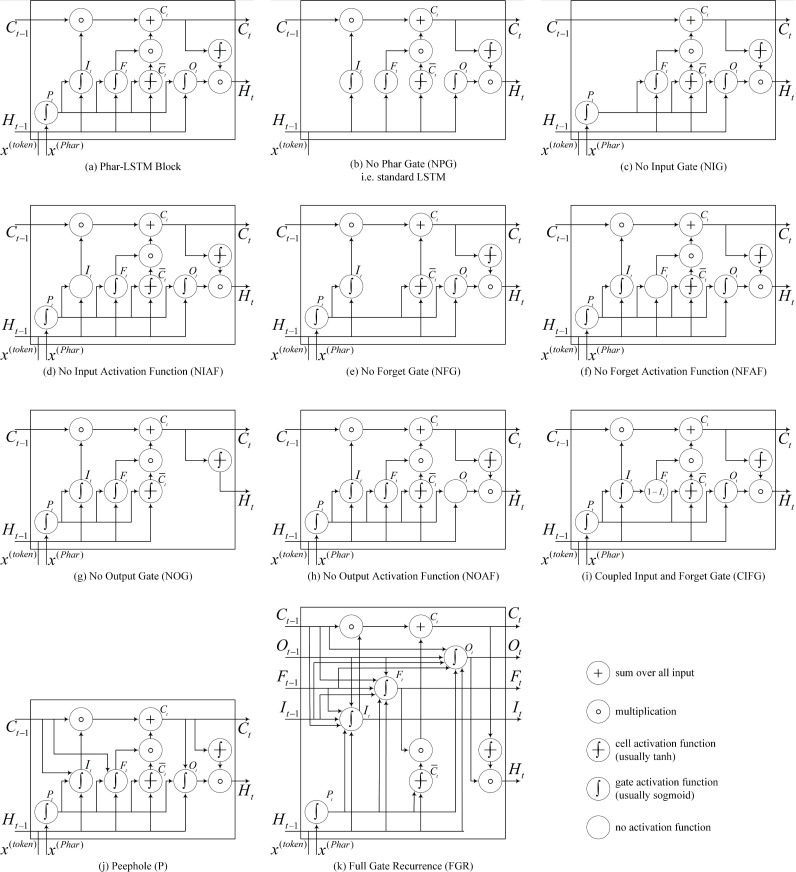
(A–K) Details of the 10 different LSTM variants.

Most previous studies tackled two tasks (*i.e.,* the coarse-grained task and fine-grained task) separately. Because multi-task learning may learn the common features of the related tasks that would benefit each task (Caruana, 1997), here, we present an LSTM-based multi-task learning scheme as shown in Fig. 3 (flowing from the bottom up). The input layer converts the raw input into the pharmacological representations. For the recurrent layer, we adopt a special Recurrent Neural Network (RNN) structure (LSTM) (Van Houdt, Mosquera & Nápoles, 2020), which can store the previous information for a long time in data processing. Note that each recurrent block of the recurrent layer can be assigned a different LSTM variant, which is illustrated in Fig. 4. The multi-task output layer feeds the common features extracted from the hidden layer into the corresponding SoftMax classifiers. We pretrain the parameters of the neural network(except the multi-task output layer) and then fine-tune in the classification stage.

The Phar-LSTM block contains four gates and a cell state, whose variation that we use here (Fig. 4A) is formulated as

(1) \begin{eqnarray*}{z}_{t}= \left( {H}_{t-1},{x}_{t}^{(\mathrm{token})} \right) ,\end{eqnarray*}

(2) \begin{eqnarray*}{P}_{t}= \left( g \left( {W}^{P}{x}^{(\mathrm{Phar})}+{b}^{P} \right) ,{z}_{t} \right) ,\end{eqnarray*}

(3) \begin{eqnarray*}{C}_{t}=g \left( {W}^{I}{P}_{t}+{b}^{I} \right) \circ {C}_{t-1}+g \left( {W}^{F}{P}_{t}+{b}^{F} \right) \circ h \left( {W}^{C}{p}_{t}+{b}^{C} \right) ,\end{eqnarray*}

(4) \begin{eqnarray*}{O}_{t}=g \left( {W}^{O}{P}_{t}+{b}^{O} \right) ,\end{eqnarray*}

(5) \begin{eqnarray*}{H}_{t}={O}_{t}\circ h \left( {C}_{t} \right) .\end{eqnarray*}

In the above, ${x}_{t}^{(\mathrm{token})}$ and ${x}_{t}^{(\mathrm{Phar})}$ represent the corresponding vectors (*i.e.,* the pharmacological representation) generated by the *t* th token using the embedding strategy (see *Embedding based pharmacological representation*). *W*^*P*^, *W*^*C*^, *W*^*I*^, *W*^*F*^, and *W*^*O*^ are the weight matrices for the pharmacological gate, the cell state, the input gate, the forget gate, and the output gate separately, and *b*^*P*^, *b*^*C*^, *b*^*I*^, *b*^*F*^, and *b*^*O*^, respectively, are the corresponding bias units. The functions *g* and *h* are activation functions. The sigmoid function is usually used as *g* for the four gates, and the tangent function is typically used as *h* for the cell state. The ∘ denotes point-wise multiplication.

### Other LSTM variants

Different structures of LSTM may influence the results; therefore, we studied the DDI extraction performance of the models with different LSTM variants. The LSTM variants can be derived by modifying the gates, activation function, and connections. The derived 10 variants are shown in Fig. 4, and the details are as follows:

(1) No pharmacological gate (NPG)

Graves & Schmidhuber (2005) originally proposed the LSTM, which is also known as vanilla LSTM. Phar-LSTM can be transformed to the vanilla LSTM by removing the pharmacological gate:

(6) \begin{eqnarray*}{C}_{t}=g \left( {W}^{I}{z}_{t}+{b}^{I} \right) \circ {C}_{t-1}+g \left( {W}^{F}{z}_{t}+{b}^{F} \right) \circ h \left( {W}^{C}{z}_{t}+{b}^{C} \right) .\end{eqnarray*}

(2) No input gate (NIG)

By removing the input gate, we obtain a lighter version of *C*_*t*_.*C*_*t*_ conveys less information to the next node:

(7) \begin{eqnarray*}{C}_{t}={C}_{t-1}+g \left( {W}^{F}{P}_{t}+{b}^{F} \right) \circ h \left( {W}^{C}{P}_{t}+{b}^{C} \right) .\end{eqnarray*}

(3) No Input Activation Function (NIAF)

By removing the activation function of *I*_*t*_, we obtain a “wilder” version of *I*_*t*_ since *I*_*t*_ is no longer confined to $ \left[ -1,1 \right] $ by the sigmoid function:

(8) \begin{eqnarray*}{C}_{t}= \left( {W}^{I}{P}_{t}+{b}^{I} \right) \circ {C}_{t-1}+g \left( {W}^{F}{P}_{t}+{b}^{F} \right) \circ h \left( {W}^{C}{P}_{t}+{b}^{C} \right) .\end{eqnarray*}

(4) No forget gate (NFG)

Gers, Schmidhuber & Cummins (2000) first proposed a variant of LSTM by adding a forget gate, which enabled the LSTM to better forget the history information. By removing the forget gate, we obtain a lighter version of *C*_*t*_. The *C*_*t*_ of NFG can remember more information because the function of the forget gate is to restrain the useless information from persisting in the history.

(9) \begin{eqnarray*}{C}_{t}=g \left( {W}^{I}{P}_{t}+{b}^{I} \right) \circ {C}_{t-1}+h \left( {W}^{C}{P}_{t}+{b}^{C} \right) .\end{eqnarray*}

(5) No forget activation function (NFAF)

Similar to the NIAF, we obtain a “wilder” version of *F*_*t*_ by removing the activation function of *F*_*t*_:

(10) \begin{eqnarray*}{C}_{t}=g \left( {W}^{I}{P}_{t}+{b}^{I} \right) \circ {C}_{t-1}+ \left( {W}^{F}{P}_{t}+{b}^{F} \right) \circ h \left( {W}^{C}{P}_{t}+{b}^{C} \right) .\end{eqnarray*}

(6) No output gate (NOG)

Similar to the NIG, by removing the output gate, we obtain a lighter version of *H*_*t*_, and *C*_*t*_ conveys less information to the next node:

(11) \begin{eqnarray*}{H}_{t}=h \left( {C}_{t} \right) .\end{eqnarray*}

(7) No output activation function (NOAF)

Similar to the NIAF, by removing the activation function of *O*_*t*_, we obtain a “wilder” version of *O*_*t*_ because *O*_*t*_ is no longer confined to $ \left[ -1,1 \right] $ by the sigmoid function:

(12) \begin{eqnarray*}{O}_{t}={W}^{O}{P}_{t}+{b}^{O}.\end{eqnarray*}

(8) Coupled input and forget gate (CIFG)

Instead of separately calculating what should be forgotten and what should be inputted as new information, the CIFG combines the two steps. The CIFG forgets only when inputting something in its place and inputs new values to the state only when forgetting something older:

(13) \begin{eqnarray*}& & {C}_{t}=g \left( {W}^{I}{P}_{t}+{b}^{I} \right) \circ {C}_{t-1}+ \left( 1-g \left( {W}^{I}{P}_{t}+{b}^{I} \right) \right) \circ h \left( {W}^{C}{P}_{t}+{b}^{C} \right) .\end{eqnarray*}

(9) Peephole (P)

Gers & Schmidhuber (2000) argued that the cell state should control the gates in order to learn precise timings. Therefore, we add connections from the cell to the gates in Phar-LSTM, which are named as Peephole, to make precise timings easier to learn:

(14) \begin{eqnarray*}& & {C}_{t}=g \left( {W}^{I} \left( {P}_{t},{C}_{t-1} \right) +{b}^{I} \right) \circ {C}_{t-1}+g \left( {W}^{F} \left( {P}_{t},{C}_{t-1} \right) +{b}^{F} \right) \circ h \left( {W}^{C}{P}_{t}+{b}^{C} \right) ,\end{eqnarray*}

(15) \begin{eqnarray*}{O}_{t}=g \left( {W}^{O} \left( {P}_{t},{C}_{t} \right) +{b}^{O} \right) .\end{eqnarray*}

(10) Full gate recurrence (FGR)

The LSTM (Van Houdt, Mosquera & Nápoles, 2020) consists of cell state and input and output gates and does not include the forget gate and peephole connections. A hybrid of real-time recurrent learning (Robinson & Fallside, 1987) and backpropagation through time (Werbos, 1988) is used for training. In this case, only the gradient of the cell state was propagated back, and the gradient for the other recurrent connections was truncated. FGR means that all the gates received recurrent inputs from the previous time step:

(16) \begin{eqnarray*}{I}_{t}=g \left( {W}^{I} \left[ \begin{array}{@{}c@{}} \displaystyle {P}_{t},\\ \displaystyle {I}_{t-1},\\ \displaystyle {F}_{t-1},\\ \displaystyle {O}_{t-1},\\ \displaystyle {C}_{t-1} \end{array} \right] +{b}^{I} \right) ,\end{eqnarray*}

(17) \begin{eqnarray*}{F}_{t}=g \left( {W}^{F} \left[ \begin{array}{@{}c@{}} \displaystyle {P}_{t},\\ \displaystyle {I}_{t-1},\\ \displaystyle {F}_{t-1},\\ \displaystyle {O}_{t-1},\\ \displaystyle {C}_{t-1} \end{array} \right] +{b}^{F} \right) ,\end{eqnarray*}

(18) \begin{eqnarray*}{O}_{t}=g \left( {W}^{O} \left[ \begin{array}{@{}c@{}} \displaystyle {P}_{t},\\ \displaystyle {I}_{t-1},\\ \displaystyle {F}_{t-1},\\ \displaystyle {O}_{t-1},\\ \displaystyle {C}_{t-1} \end{array} \right] +{b}^{O} \right) .\end{eqnarray*}

### Classification and training

We use the SoftMax classifier for classification. Let *k* denote the number of DDI types. The output $o\in {R}^{ \left\vert k \right\vert }$ is the probabilities of each class to which *S* belongs.

(19) \begin{eqnarray*}y=\arg \nolimits \max \nolimits \left( \frac{1}{\exp \nolimits \left( W{H}_{n}+b \right) } \left[ \begin{array}{@{}c@{}} \displaystyle \exp \nolimits \left( {W}_{1}{H}_{n}+b \right) \\ \displaystyle \exp \nolimits \left( {W}_{2}{H}_{n}+b \right) \\ \displaystyle \cdots \\ \displaystyle \exp \nolimits \left( {W}_{k}{H}_{n}+b \right) \end{array} \right] \right) .\end{eqnarray*}

We use the cross-entropy (De Boer et al., 2005) cost function and ridge regularization (Hoerl & Kennard, 1970) as the optimization objective. For the *i* th instance, *y*^(*i*)^ denotes the output. The cross-entropy cost is

(20) \begin{eqnarray*}J=- \left( {\mathop{\sum \nolimits }\nolimits }_{j=1}^{k}1 \left\{ {y}^{(i)}=j \right\} \log \nolimits \frac{\exp \nolimits \left( {W}_{j}{H}_{n}+b \right) }{{\mathop{\sum \nolimits }\nolimits }_{l=1}^{k}\exp \nolimits \left( W{H}_{n}+b \right) } \right) + \frac{\lambda }{2} { \left\| W \right\| }^{2},\end{eqnarray*}

where $1 \left\{ \blacksquare \right\} $ is the indicator function, such that $1 \left\{ \text{a true statement} \right\} =1$ and $1 \left\{ \text{a false statement} \right\} =0$. We optimize the parameters of the objective function *J* with Rmsprop (Chowdhury & Lavelli, 2011), which is a variant of mini-batch stochastic gradient descent. During each training step, the gradient of *J* is calculated. Then, all the parameters are adjusted according to the gradient. After the end of training, we have a model that is able to predict two drugs’ interactions when a sentence about these drugs is given.

## Results and Discussion

To evaluate the performance of our method for DDI extraction, extensive experiments are conducted to compare the Phar-LSTM approach with different variants and other state-of-art methods on the DDIExtraction 2011 and DDIExtraction 2013 datasets. The setup of the experiments is designed to be as simple as possible to make the comparisons fair.

### Evaluation metrics

In this section, we describe the evaluation metrics used in our experiments. For the DDIExtraction 2011 and DDIExtraction 2013 corpuses, Precision (P), Recall (R), *F*-score (F), and Accuracy (Acc) are widely used as the evaluation metrics (Asada, Miwa & Sasaki, 2023). Since the DDIExtraction 2013 corpus is a multi-class classification problem, we adopt the micro-average and macro-average strategy to score the overall performance on the five classes.

To obtain the *F*-score, the contingency table (or confusion matrix) is built first, in which each row of the matrix represents the instances in a predicted class and each column represents the instances in an actual class. The contingency table enables us to obtain the true positive (TP), false positive (FP), false negative (FN), and true negative (TN). Based on that, the precision, recall, *F*-score, and accuracy can be defined as follows:

(21) \begin{eqnarray*}P= \frac{\text{TP}}{\text{TP}+\text{FP}} ,\end{eqnarray*}

(22) \begin{eqnarray*}R= \frac{\text{TP}}{\text{TP}+\text{FN}} ,\end{eqnarray*}

(23) \begin{eqnarray*}F=2\times \frac{P\times R}{P+R} ,\end{eqnarray*}

(24) \begin{eqnarray*}\text{Acc}= \frac{\text{TP}+\text{TN}}{\text{TP}+\text{TN}+\text{FP}+\text{FN}} .\end{eqnarray*}

For the DDIExtraction 2013 corpus, there are five P, R, and *F*-score values for each class since there are five different classes (the five DDI types: ADVICE, EFFECT, MECHANISM, INT, and NEGATIVE). Each DDI type is evaluated separately. Moreover, to measure the overall performance, two commonly used metrics, *i.e.,* micro-averaged *F*-score (CLA) and macro-averaged *F*-scores (MAVG), are calculated. The CLA is calculated by constructing a global contingency table and then calculating the precision and recall, and the MAVG is calculated by first calculating the precision and recall for each type and then taking the average of those results.

To evaluate the scalability of our method, we propose a metric to evaluate the performance gap of the models between the two corpuses under the assumption that if a model has good performance in scalability, it would not only have a high average *F*-score but also have less variance. For instance, the *F*-scores of model A are 0.65 and 0.65 on the DDIExtraction 2011 and 2013 corpuses, respectively, and those of model B are 0.60 and 0.70. Although the average *F*-scores of model A and model B are both 0.65, model A would be considered to have better scalability than model B. Based on this, the metric can be defined using the 1-standard deviation of *F*-scores as

(25) \begin{eqnarray*}1-\sigma =1-\sqrt{\begin{array}{@{}c@{}} \displaystyle \frac{1}{2} { \left( {F}_{2011}- \frac{{F}_{2011}+{F}_{2013}}{2} \right) }^{2}+ \frac{1}{2} { \left( {F}_{2013}- \frac{{F}_{2011}+{F}_{2013}}{2} \right) }^{2} \end{array}}.\end{eqnarray*}

To evaluate the consistency, the training process for 200 epochs of each learning model is shown as a boxplot, and Welch’s *t*-test at a significance level of alpha = 0.05 was used to determine whether the mean test set performance of a learning model was significantly different from that of Phar-LSTM.

To evaluate the reproducibility, we first ran the training process for 10 times using different random seeds and obtained the boxplot of the overall training process to show the gap between each run. Based on the boxplot, we further calculated the sum of the variance of the *F*-scores of each epoch and the sum of the standard deviation of the *F*-scores of each epoch to measure the differences among the 10 runs.

### Hyperparameter settings

Based on previous research and experience, the Phar-LSTMs were trained by an RMSprop optimizer with a loss function of cross entropy and a learning rate of 0.001. Dropout layers were added to each of the embedding layers and hidden layers with a ratio of 0.2. For each run, the number of training epochs were 200 and the batch sizes were 32. All the experiments were run on GeForce GTX-1080 and took 9.3 h on average to complete.

### Scalability

To evaluate the scalability of our method, experiments were conducted on both the DDIExtraction 2011 and DDIExtraction 2013 datasets. Before 2013, most studies were evaluated on the DDIExtraction 2011 dataset. After 2013, most research has focused on evaluating the methods on the DDIExtraction 2013 dataset. As far as we know, few methods (Björne, Kaewphan & Salakoski, 2013; Jiang, Gu & Jiang, 2017; Thomas et al., 2013) have been evaluated on both datasets.

We first compared the performance of our scheme with the traditional methods as well as the deep learning-based method on the DDIExtraction 2011 dataset. The results are shown in Table 1. The traditional methods are typically based on manually extracted features or kernels. For example, Björne et al. (2011b) leveraged many syntactic-based features, including tokens, dependency types, POS tags, text, and stems. Similarly, Thomas et al. (2013) combined an all-path-graph kernel, a shallow linguistic kernel, and a k-band shortest path spectrum kernel, which were all derived from syntactic analysis. Other methods such as FBK-HLT (Chowdhury et al., 2011) and LIMSI-FBK (Björne et al., 2011a) used either features or kernels or both.

**Table 1:** Scalability comparison of different methods on the DDIExtraction 2011 dataset (the bold indicating the best value on the corresponding metric).

Method	Evaluation metrics							
	TP	FP	FN	TN	P	R	F	Acc
WBI (Thomas et al., 2013)	543	354	212	5,914	0.6045	0.7192	0.6574	0.9194
LIMSI-FBK (Björne et al., 2011a)	532	376	223	5,895	0.5859	0.7046	0.6398	0.9147
FBK-HLT (Chowdhury et al., 2011)	529	377	226	5,894	0.5839	0.7007	0.6370	0.9142
Uturku (Björne, Kaewphan & Salakoski, 2013)	520	376	235	5,895	0.5804	0.6887	0.6299	0.9130
LIMSI-CNRS (Segura-Bedmar, Martínez Fernández & Sánchez Cisneros, 2011)	490	398	265	5,873	0.5518	0.6490	0.5965	0.9056
BNBNLEL (Segura-Bedmar, Martínez Fernández & Sánchez Cisneros, 2011)	420	**266**	335	**6,005**	0.6122	0.5563	0.5829	0.9145
Skeleton-LSTM (Jiang, Gu & Jiang, 2017)	550	320	205	5951	0.6322	0.7285	0.6769	0.9253
Phar-LSTM	**559**	311	**196**	5,960	**0.6425**	**0.7404**	**0.6880**	**0.9278**

These features and kernels are highly dependent on third-party tools such as syntactic parsing, which makes the method sensitive to the quality of the parsing results and the expertise of researchers in designing features or kernels. Therefore, although the heuristic idea of using features and kernels can be helpful to other researchers, the models themselves may not have good scalability.

It can be observed from Table 1 that the Phar-LSTM scheme achieved the best performance, with the *F*-score of 0.6880. Another deep learning-based method, Skeleton-LSTM (Jiang, Gu & Jiang, 2017) (*F*-score: 0.6769), also performed significantly better than other traditional methods, which illustrates the superiority of deep learning-based methods for the coarse-grained task of DDI extraction.

Table 2 shows the results of our scheme in comparison with the baselines on the DDIExtraction 2013 dataset. From Table 2, we can observe that Phar-LSTM achieved the best performance in terms of MAVG and CLA (0.708 and 0.716, respectively). Skeleton-LSTM (Jiang, Gu & Jiang, 2017) had similar performance as Phar-LSTM and performed significantly better than other baselines. One posssible reason may be that Skeleton-LSTM and Phar-LSTM both use the end-end-learning framework (*i.e.,* feed the raw input into the neural network and produce the output directly), which would capture some latent features because the features are automatically extracted by the neural network rather than by third-party tools.

**Table 2:** Scalability comparison of Phar-LSTM with other methods on the DDIExtraction 2013 dataset (the best value on each metric is highlighted in bold).

Method	Evaluation metrics						
	NEG	MEC	EFF	ADV	INT	MAVG	CLA
FBK-irst (Chowdhury & Lavelli, 2013)	**0.8**	0.679	0.628	0.692	**0.547**	0.648	0.651
NIL_UCM (Bokharaeian & Díaz, 2013)	0.588	0.515	0.489	0.613	0.427	0.535	0.517
SCAI (Bobić, Fluck & Hofmann, 2013)	0.683	0.441	0.440	0.559	0.021	0.448	0.452
UC3M (Sánchez Cisneros, 2013)	0.676	0.480	0.547	0.575	0.500	0.534	0.529
UCOLORADO SOM (Hailu, Hunter & Cohen, 2013)	0.504	0.361	0.311	0.381	0.333	0.407	0.334
Uturku (Björne, Kaewphan & Salakoski, 2013)	0.696	0.582	0.600	0.630	0.507	0.587	0.594
UWM-TRIADS (Rastegar-Mojarad, Boyce & Prasad, 2013)	0.599	0.446	0.449	0.532	0.421	0.472	0.470
WBI (Thomas et al., 2013)	0.736	0.602	0.604	0.618	0.516	0.588	0.599
Kim (Kim et al., 2015)	0.775	0.693	0.662	0.725	0.483	–	0.670
CNN (Liu et al., 2016)	–	–	–	–	–	–	0.698
Attention-CNN (Asada, Miwa & Sasaki, 2017)	–	0.695	0.681	0.773	0.455	–	0.691
One-stage SCNN (Zhao et al., 2016)	–	–	–	–	–	–	0.670
Two-stage SCNN (Zhao et al., 2016)	–	–	–	–	–	–	0.686
SVM+LSTM (Huang et al., 2017)	–	**0.738**	**0.720**	0.715	0.549	0.690	–
Skeleton-LSTM (Jiang, Gu & Jiang, 2017)	0.795	0.725	0.701	0.788	0.484	0.707	0.714
AB-LSTM (Sahu & Anand, 2018)	–	0.681	0.683	0.697	0.542	0.650	–
Joint AB-LSTM (Sahu & Anand, 2018)	–	0.723	0.655	**0.803**	0.441	0.655	–
Phar-LSTM	0.795	0.726	0.699	0.789	0.482	**0.708**	**0.716**

To further evaluate the scalability of our scheme, three baselines were chosen for comparing with our defined metric, and the results are shown in Table 3. Note that many methods developed for addressing the coarse-grained DDI extraction task may not be applicable for the fine-grained task of DDI extraction. It can be observed in Table 3 that the Phar-LSTM scheme achieved the highest (1 − *σ*) value (0.986) among all methods, which demonstrates the scalability of our method.

**Table 3:** Scalability comparison of Phar-LSTM with other methods on the two datasets (the bold denoting the best value on the corresponding metric).

Method	Evaluation metrics		
	*F*_2011_	*F*_2013_	1 − *σ*
WBI (Thomas et al., 2013)	0.6574	0.599	0.9708
Uturku (Björne, Kaewphan & Salakoski, 2013)	0.6299	0.594	0.9821
Skeleton-LSTM (Jiang, Gu & Jiang, 2017)	0.6769	0.714	0.9810
Phar-LSTM	**0.6880**	**0.716**	**0.9860**

### Consistency

**Figure 5:**
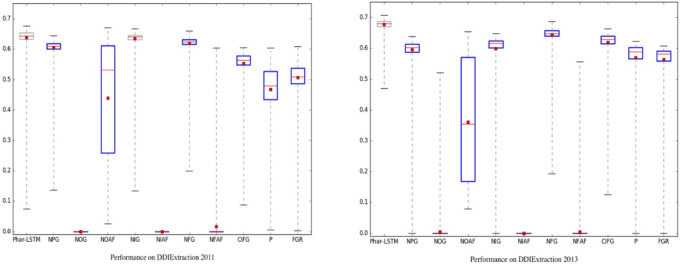
Consistency comparison of Phar-LSTM with 10 variants at a significance level of alpha = 0.05 (Welch’s *t*-test) on the DDIExtraction 2011 dataset and DDIExtraction 2013 dataset.

To evaluate the consistency of the Phar-LSTM scheme, we compared Phar-LSTM with 10 different variants of LSTM, in which the number of epochs for each variant were set to 200. Welch’s *t*-test at a significance level of alpha = 0.05 was used to determine whether the performance of each variant was significantly different from another. A summary of the results of the different methods with 200 epochs is shown in Fig. 5. The boxplots of the variants that differ significantly from Phar-LSTM are highlighted in blue.

It can be observed from Fig. 5 that Phar-LSTM generally achieved the best performance on both datasets. Moreover, the *F*-scores of Phar-LSTM for most epochs were relatively stable, which indicates the consistency of our method. Another observation based on Fig. 5 is that removing the output gate (NOG) or the activation functions (NOAF, NIAF, and NFAF) significantly hurt the performance on the two datasets. The ability to output information and the activation of the perceptron appear to be critical for the LSTM architecture. This is probably because the output value of the hidden layers cannot be constrained without the activation function and therefore fails to train the parameters. If the output gate is removed from the LSTM unit, although ${x}_{t}^{(\text{token})}$ and ${x}_{t}^{(\text{Phar})}$ can be integrated to the hidden layer *H*_*t*_ by *C*_*t*_, the original information of and ${x}_{t}^{(\text{token})}$ and ${x}_{t}^{(\text{Phar})}$ is diluted during the calculation of *H*_*t*_. And then, the final SoftMax regression built on the hidden layer of the last unit captures little information of the input, which leads to the failure of training.

On the contrary, although removing the input gate (NIG) or forget gate (NFG) or coupling them into one gate (CIFG) can decrease the *F*-score, comparing with the NOG, the input information is still integrated through the other gate (*e.g.*, the forget gate for the NIG and the input gate for the NFG). Therefore, the parameters can be trained successfully.

Similarly, removing the pharmacological gate (NPG) generally decreases the *F*-score more than the NIG, NFG, and CIFG do. This illustrates that the pharmacological gate contains important information to represent the DDI than other gates do. This proves that the Phar-LSTM scheme indeed improved the DDI extraction performance by incorporating the pharmacological gate.

Both adding the Peephole (P) and the full gate recurrence (FGR) decrease the performance while increasing the computational complexity. We generally advise against using them for DDI extraction.

### Reproducibility

**Figure 6:**
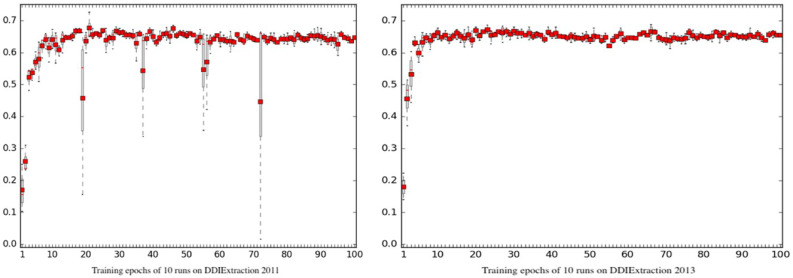
Reproducibility of Phar-LSTM with different epochs for 10 runs on the DDIExtraction 2011 dataset and DDIExtraction 2013 dataset.

Due to the random seed mechanism and the implementation of the GPU training architecture, the training process is usually unreproducible. To evaluate the reproducibility of Phar-LSTM, we ran our scheme for many times to check the differences of the outputs. The boxplots of Phar-LSTM’s performance for 10 runs with different epochs on the two datasets are shown in Fig. 6, from which we can see that the performances of Phar-LSTM for most epochs are close.

Some specific epochs can be observed for DDIExtraction 2011, such as the 19th, 37th, 55th, 56th, and 72th epochs. For these specific epochs, the performance of different runs differed from each other. However, there were no such specific epochs for DDIExtraction 2013. One possible reason may be the data distribution. DDIExtraction 2011 is smaller, and the annotation strategy is different from that of DDIExtraction 2013. Researchers should be aware of these specific epochs. The best way to check the model is using a validation set. By conducting experiments on the validation set and drawing the boxplot of the learning curve of different runs, researchers can easily find these specific epochs and improve their extraction system.

Another finding is that both the climb stages (0 < epoch < 10) of DDIExtraction 2011 and DDIExtraction 2013 blurred. The reason is that the initial random states of the parameters are different, which may cause the performances to differ during the climb stage. However, after the climb stage, the performances with the following epochs are much closer. This means that the Phar-LSTM scheme can adapt to different initial random seeds.

To further compare the reproducibility of Phar-LSTM with other variants, we summed the variance and the standard deviation of each epoch for the 10 runs. The metrics (variance and standard deviation) can indicate the overall reproducibility. From Table 4, we can see that Phar-LSTM, NOG, NIAF, and NFAF had better scores than the other models, and the conclusion is consistent with Fig. 5. However, from Fig. 5, we can see that the NOG, NIAF and NFAF had poor *F*-scores. Although the results of the three models can be reproduced easily, the value of the three models is low. Phar-LSTM reaches a good balance between high reproducibility and high *F*-score.

**Table 4:** Reproducibility comparison of Phar-LSTM with other methods on DDIExtraction 2013.

Metric	Different approaches									
	Phar-LSTM	NPG	NOG	NOAF	NIG	NIAF	NFG	NFAF	CIFG	*P*
Variance^2011^	0.2219	0.5625	0.1283	12.7918	0.4409	0.1380	0.3634	0.1752	0.3967	0.6529
Standard deviation^2011^	2.8032	5.6749	1.6493	150.108	4.1166	1.2839	3.4471	2.1577	4.5873	6.3908
Variance^2013^	0.0222	0.03979	0.0146	4.1293	0.0631	0.0164	0.0364	0.0193	0.0413	0.1695
Standard deviation^2013^	1.3934	2.7890	0.7589	48.3608	3.8259	0.7765	1.8223	0.8131	2.4978	3.6656

## Conclusions and Future Work

In this study, we proposed a pharmacological representation-based LSTM network to extract DDIs from the biomedical literature. Different from previous studies, we adopted a new embedding strategy, in which the documents were represented as a sequence of word embeddings and target drug relative information embeddings, called pharmacological representations. An LSTM-based multi-task learning scheme was introduced to extract features of the pharmacological representations from two related DDI extraction tasks (*i.e.,* the coarse-grained task and fine-grained task). Experimental results showed that our scheme outperformed other state-of-the-art methods on both DDIExtraction 2011 (the coarse-grained task) and DDIExtraction 2013 (the fine-grained task). The scalability, consistency, and reproducibility of our scheme were evaluated on both datasets, and the results demonstrated the relatively superior performance of our method in these aspects.

In our forthcoming work, we will address the existing issues to enhance the prediction of DDI events. First, we will extend our method to other biomedical relative extraction tasks, such as protein–protein interaction extraction and chemical–disease interaction extraction. Second, there are insufficient interactions for certain events, and we will explore data augmentation techniques to expand the event dataset.

## Supplemental Information

10.7717/peerj.16606/supp-1 Supplemental Information 1 The datasets and source codes leveraged and/or analyzed in this study

## Additional Information and Declarations

### Data Availability

The following information was supplied regarding data availability: The datasets and source codes are available at Zenodo: Huang Mingqing, Jiang Zhenchao, & Guo Shun. (2023). Data and Codes of “A Pharmacological Representation-based LSTM Network for Drug–Drug Interaction Extraction” [Data set]. Zenodo. https://doi.org/10.5281/zenodo.8249384.
