# Supplementary material for: Phar-LSTM: a pharmacological representation-based LSTM network for drug–drug interaction extraction
Source: PeerJ. 2023 Dec 14;11:e16606. doi: 10.7717/peerj.16606 (PMC10725669; doi:10.7717/peerj.16606)
Supplement: Supplemental Information 1 [file peerj-11-16606-s001.zip › Code_DDIEClassifier.ipynb]

phar-LSTM/DDIE\_classifier.ipynb at master · kukumayas/phar-LSTM · GitHub


Skip to content


Sign up

- Product

  - Features
  - Mobile
  - Actions
  - Codespaces
  - Copilot
  - Packages
  - Security
  - Code review
  - Issues
  - Integrations
  - GitHub Sponsors
  - Customer stories
- Team
- Enterprise
- Explore

  - Explore GitHub
  - Learn and contribute
  - Topics
  - Collections
  - Trending
  - Skills
  - GitHub Sponsors
  - Open source guides
  - Connect with others
  - The ReadME Project
  - Events
  - Community forum
  - GitHub Education
  - GitHub Stars program
- Marketplace
- Pricing

  - Plans
  - Compare plans
  - Contact Sales
  - Education

- In this repository

  All GitHub
  ↵

  Jump to
  ↵

- No suggested jump to results

- In this repository

  All GitHub
  ↵

  Jump to
  ↵
- In this user

  All GitHub
  ↵

  Jump to
  ↵
- In this repository

  All GitHub
  ↵

  Jump to
  ↵

Sign in

Sign up

{{ message }}

kukumayas
/
**phar-LSTM**
Public

- Notifications
- Fork
  0
- Star
   0

- Code
- Issues
  0
- Pull requests
  0
- Actions
- Projects
  0
- Wiki
- Security
- Insights

More

- Code
- Issues
- Pull requests
- Actions
- Projects
- Wiki
- Security
- Insights

Permalink

master

Switch branches/tags


Branches
Tags

Could not load branches


Nothing to show

{{ refName }}
default
View all branches

Could not load tags


Nothing to show


{{ refName }}
default
View all tags

## phar-LSTM/**DDIE\_classifier.ipynb**

 Go to file

 

- Go to file
  T
- Go to line
  L
- Copy path
- Copy permalink

This commit does not belong to any branch on this repository, and may belong to a fork outside of the repository.

kukumayas

Add files via upload

Latest commit
b116c18
Nov 23, 2019


**History**

**1**
contributor


### Users who have contributed to this file

1312 lines (1312 sloc)
62.2 KB

Raw
  Blame

Edit this file

E


Open in GitHub Desktop

- Open with Desktop
- View raw
- Copy raw contents
   Copy raw contents

   Copy raw contents

   Copy raw contents
- View blame

Sorry, something went wrong. Reload?

Sorry, we cannot display this file.

Sorry, this file is invalid so it cannot be displayed.

Viewer requires iframe.

 Go

## Footer

© 2022 GitHub, Inc.

### Footer navigation

- Terms
- Privacy
- Security
- Status
- Docs
- Contact GitHub
- Pricing
- API
- Training
- Blog
- About

You can’t perform that action at this time.

You signed in with another tab or window. Reload to refresh your session.
You signed out in another tab or window. Reload to refresh your session.
